# Anatomic predictor of severe prosthesis malposition following transcatheter aortic valve replacement with self- expandable Venus-A Valve among pure aortic regurgitation: A multicenter retrospective study

**DOI:** 10.3389/fcvm.2022.1002071

**Published:** 2022-12-08

**Authors:** Yong Wang, Shiyong Yu, Dehui Qian, Jie Li, Zhenfei Fang, Wei Cheng, Xiaoqing Li, Ting Liu, Ying Zeng, Hongmei Xia, Jun Jin

**Affiliations:** ^1^Department of Cardiology, Institute of Cardiovascular Research, Xinqiao Hospital, Army Medical University, Chongqing, China; ^2^Guangdong Cardiovascular Institute, Guangdong Provincial People’s Hospital, Guangdong Academy of Medical Sciences, Guangzhou, China; ^3^Department of Cardiovascular Medicine, The Second Xiangya Hospital, Central South University, Changsha, China; ^4^Department of Cardiac Surgery, Southwest Hospital, Army Medical University, Chongqing, China; ^5^Department of Ultrasound, Xinqiao Hospital, Army Medical University, Chongqing, China

**Keywords:** pure native aortic regurgitation, computed tomography, malposition, self- expandable, transcatheter aortic valve replacement

## Abstract

**Background:**

Transcatheter aortic valve replacement (TAVR) in the treatment of patients with pure native aortic valve regurgitation (NAVR) has been based on the “off-label” indications, while the absence of aortic valve calcification and difficulty in anchoring was found to significantly increase the risk of prosthesis malposition. The aim of this study was to explore the anatomical predictors of severe prosthesis malposition following TAVR with the self-expandable Venus-A Valve among patients with NAVR.

**Methods:**

A total of 62 patients with NAVR who underwent TAVR with Venus-A Valve at four Chinese clinical centers were retrospectively observed. The clinical features, aortic multidetector computed tomography (MDCT) data, and clinical outcomes were compared between non-/mild malposition and severe malposition groups. Univariate logistic regression analysis was used to identify the risk factors of severe prosthesis malposition, and the receiver operating characteristic (ROC) curve was used to explore the predictive value of the risk factors.

**Results:**

Valve migration to ascending aortic direction occurred in 1 patient, and the remaining 61 patients (including 19 severe malposition cases and 42 non-/mild malposition cases) were included in the analysis. The diameter and height of the sinotubular junction (STJ) and STJ cover index (STJCI, calculated as 100%*STJ diameter/nominal prosthesis crown diameter) were all greater in the severe malposition group (all *p* < 0.05). Logistic regression showed that STJ diameter (OR = 1.23, 95% CI 1.04–1.47, *p* = 0.017), STJ height (OR = 1.24, 95% CI 1.04–1.47, *p* = 0.017), and STJCI (OR = 1.08, 95% CI 1.01–1.16, *p* = 0.032) were potential predictors for severe prosthesis malposition. The area under the ROC curve was 0.72 (95% CI 0.58–0.85, *p* = 0.008) for STJ diameter, 0.70 (95% CI 0.55–0.86, *p* = 0.012) for STJ height, and 0.69 (95% CI 0.55–0.83, *p* = 0.017) for STJCI, respectively. The cutoff value was 33.2 mm for STJ diameter (sensitivity was 84.2% and specificity was 65.8%), 24.1 mm for STJ height (sensitivity was 57.9% and specificity was 87.8%), and 81.0% for STJCI (sensitivity was 68.4% and specificity was 68.3%), respectively.

**Conclusion:**

Larger and higher STJ, as well as greater STJ to valve crown diameter ratio, may help identify patients at high risk for severe prosthesis malposition among patients with NAVR undergoing TAVR with Venus-A prosthesis valve.

## Introduction

Compared to Western countries, aortic regurgitation (AR) is more prevalent than aortic stenosis among the elderly in China (1). As transcatheter aortic valve replacement (TAVR) came into use for lower-risk patients with severe aortic stenosis, and “off-label” indications for TAVR in the treatment of patients with pure native aortic valve regurgitation (NAVR) have also been explored (2–4). When left untreated, NAVR is associated with high mortality risk. There are also many patients at high surgical risk or unwilling to undergo surgery, for whom less invasive trans-catheter treatment continues to present a feasible option. However, the absence of aortic valve calcification and difficulty in anchoring the prosthesis valve significantly increase the risk of valve migration/embolization, additional valve implantation, and significant residual regurgitation (2, 4).

The Valve Academic Research Consortium-3 (VARC-3) defines prosthesis malposition as valve migration, valve embolization, and ectopic valve deployment (5). In a previous study, the incidence of device malposition has been reported to amount to 33.0% using early-generation devices (2). Furthermore, the incidence of residual AR and the need for implanting a second valve (valve-in-valve procedures) were found to remain high in the NAVR population who received TAVR (6, 7). Several devices have shown favorable results in NAVR, such as JenaValve THV (JenaValve Technology, Inc., Irvine, CA, USA) and Acurate neo (Boston Scientific, Marlborough MA, USA), while none of which were approved for use in Mainland China. Transapical J-valve (JieCheng Medical Technology, Suzhou, China) has been verified as safe and effective for use in patients with NAVR (8); however, transfemoral access TAVR continues to be the first choice instead of the transapical approach. Given that there are currently no suitable artificial transcatheter heart valves available for NAVR, and there are a large number of patients requiring treatment who are unsuitable or unwilling to undergo surgery, identifying the high-risk anatomic feature of prosthesis malposition could help with the selection of more suitable NAVR candidates for transfemoral TAVR.

In their study, Li et al. (9) revealed that conical left ventricular outflow tract and tall aortic sinuses were strong predictors of prosthesis malposition during self-expandable TAVR in patients with aortic stenosis. However, there is still limited data on anatomic risk factors for prosthesis malposition in patients with NAVR, especially among those implanted with the most widely used Venus-A Valve (Venus MedTech, Hangzhou, China) in China. Therefore, considering the different anchoring conditions of patients with NAVR, we aimed to explore the anatomical predictors of prosthesis malposition following TAVR with the self-expandable Venus-A Valve among Chinese patients with NAVR.

## Materials and methods

### Study population and design

A total of 62 consecutive patients with symptomatic severe pure NAVR who underwent TAVR using a self-expandable Venus-A Valve at one of the four Chinese centers between January 2019 and December 2021 were enrolled in this multicenter, retrospective study. All four experienced centers performed more than 100 TAVR cases per year. Venus-A Valve is the first approved transcatheter heart prosthesis valve and the most widely used one in Mainland China. The design characteristics of Venus-A Valve have been previously reported in detail (9). The second-generation Venus-A Plus Valve is resheathable and morphologically consistent with the first-generation valve (10). Patients with high/prohibitive surgical risk or those who rejected surgery were considered eligible candidates, and those with aortic stenosis defined as a peak aortic jet velocity ≥ 250 cm/s or mean transvalvular gradient ≥ 20 mmHg were excluded (11). The indication for TAVR was discussed by each heart team, and the size of the prostheses was independently determined by the individual centers based on aortic root multidetector computed tomography (MDCT). Lunderquist extra-stiff wire was used concerning its appropriate stiffness. The valve size selection and the decision on whether to implant an additional prosthesis valve were all individually decided in each heart center.

Among the 62 patients, there was 1 case of valve embolization to ascending aortic direction due to slender ascending aorta (AA) and narrow sinotubular junction (STJ) [STJ diameter was 27.2 mm, STJ cover index (STJCI) was 72.5%, and STJ height was 23.0 mm]. Given the contrasting anatomic features of aortic root among patients with upward and downward migration, there was only one case in the aortic migration group; therefore, only the downward migration patients were introduced in statistical analysis, resulting in 61 cases included in the final analysis. The patient flowchart is shown in Supplementary Figure 1, and description of surgical risk detail was shown in Supplementary Figure 2.

This study was approved by the Research Ethics Committee of the Second Affiliated Hospital of Army Military Medical University, and informed consent was waived because of the retrospective design.

### Data collection

**FIGURE 1:**
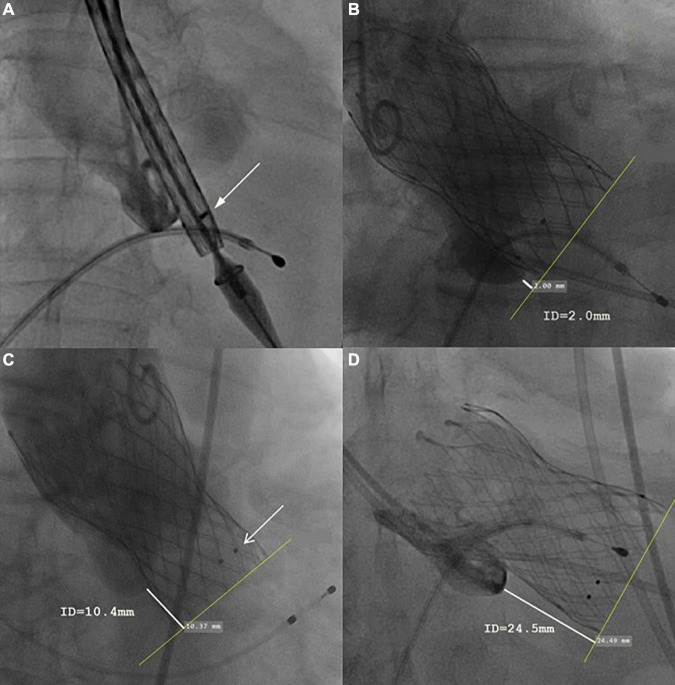
Representative case of each group. **(A)** The initial position of the prosthesis. **(B)** In optimal implantation, the implantation depth (measured as the distance from the native aortic annulus plane on the side of the non-coronary cusp to the most proximal edge of the implanted prosthesis) was 2.4 mm. **(C)** In the mild malposition case, the implantation depth was 10.4 mm, no perivalvular leakage or residual stenosis was found. The three black dots indicated by the white arrow were 5 mm from the proximal edge. **(D)** In severe malposition cases, the prosthesis migrated toward the ventricular direction during release, causing very deep implantation (implantation depth = 24.5 mm). Severe residual regurgitation was found and then a second Venus-A Valve was implanted (valve-in-valve TAVR).

Baseline clinical information, echocardiographic and MDCT data, as well as procedural data and postprocedure 30-day clinical follow-up data were collected. All patients underwent echocardiography and electrocardiography before discharge and at a 30-day follow-up. Clinical events and 30-day endpoints were all recorded according to VARC-3 criteria. Impaired anterior mitral leaflet (AML) movement was defined as significant interference with the prosthesis frame and mitral valve, thus leading to limited AML movement shown by echocardiography. The valve implantation depth was measured as the distance from the native aortic annulus plane on the side of the non-coronary cusp (or right cusp for bicuspid) to the most proximal edge of the implanted prosthesis by an instant angiogram after implantation. The recommended implantation depth for the Venus-A prosthesis was 4–8 mm below the aortic annulus. Three marker points above 5 mm from the proximal edge were designed for identifying the implantation depth during device delivering (Figures 1A,C, shown by white arrow).

### Multidetector computed tomography

Multidetector computed tomography data were retrospectively analyzed using 3mensio software (Pie Medical, Bilthoven, Netherlands) by two independent researchers who were blinded to all other clinical data. Inconsistencies were resolved by measuring again and consulting a local experienced interventional cardiologist. The aortic root structure was measured by the 40% systolic phase. Perimeter-derived diameter for annulus and average diameter of the left ventricular outflow tract, sinus of Valsalva, STJ, and AA were measured, respectively. STJ height was measured on the central line between STJ and annulus dimension automatically. The aortic valve calcification volume was automatically measured with a calcification threshold set at 850 HU. The oversize valve ratio was calculated as 100%*[(prosthesis size − annulus diameter)/annulus diameter − 1]. The STJCI was calculated as 100%*STJ diameter/nominal prosthesis crown diameter.

### Grouping

**FIGURE 2:**
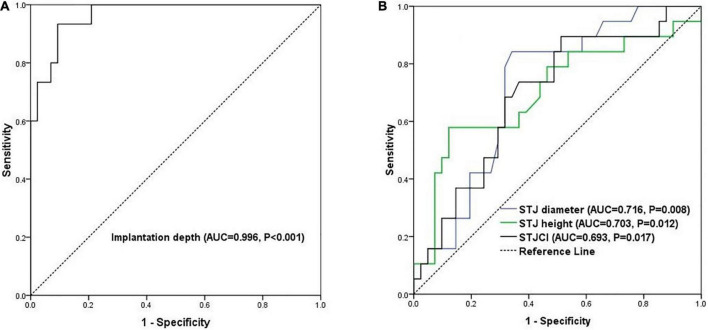
The ROC curves of **(A)** prediction of hemodynamically relevant consequences by implantation depth and **(B)** prediction of severe malposition by the three STJ indicators. AUC, area under the curve; ROC, receiver operating characteristic.

Patients were divided into an optimal position group, mild malposition group, and severe malposition group based on the modified VARC-3 criteria (Figure 1). Optimal position referred to patients with implantation depth ranging from 0 to 8.0 mm. Mild malposition was defined as >8.0 mm but with acceptable implantation depth. Severe malposition referred to very deep implantation that is prone to cause hemodynamically relevant consequences (residual transvalvular gradient ≥ 20 mmHg or more than moderate paravalvular regurgitation). To define mild and severe malposition groups, the receiver operating characteristic (ROC) curve was then used to explore the optimal threshold of implantation depth. As a result, 15.0 mm was shown to be a good cutoff value to predict residual stenosis or more than moderate paravalvular regurgitation (area under the ROC curve, AUC, = 0.996, 95% CI 0.93–1.01, *p* < 0.001, Figure 2A). Given the similar aortic root anatomic construction and clinical outcomes of the optimal implantation group and mild malposition group (Supplementary Tables 1, 2), we classified them as the non-/mild malposition group. Comparisons were made between the non-/mid malposition group and the severe malposition group.

### Statistical analysis

Continuous variables with normal distribution are expressed as mean ± standard deviation; those with skewed distribution are expressed as median (lower and upper quartile), while categorical variables are reported as numbers (percentages). The independent-sample *t*-test or the Mann–Whitney *U*-test was used to compare the means between two groups, and the chi-square test or Fisher’s exact test for categorical variables. The clinical, anatomic, and procedural indicators, which were regarded as candidate risk factors for severe valve malposition, are listed in Tables 1–3. Variables with *p* < 0.10 in inter-group comparisons or anatomical variables of interest were entered into the binary logistic regression model. Given the small sample size, only univariate analysis was performed in this study. ROC curve was used to analyze each predictor’s discriminative performance and identify the optimal cutoff value. All statistical tests were two-tailed, with *p* < 0.05 being considered statistically significant. Statistical analyses were performed using SPSS (version 26.0; Chicago, IL, USA).

**TABLE 1:** Baseline characteristics of study population.

	Total population (*n* = 61)	Severe malposition (*n* = 19)	Non/mild malposition (*n* = 42)	*P*-value
**Clinical data**				
Age, years	72.8 ± 6.7	72.5 ± 7.4	73.0 ± 6.4	0.778
Male gender	38 (62.3)	15 (78.9)	23 (54.8)	0.071
BMI, Kg/m^2^	23.3 ± 4.3	23.0 ± 3.3	23.5 ± 4.7	0.700
STS score, %	5.8 ± 1.8	6.0 ± 2.0	5.7 ± 1.8	0.683
Hypertension	41 (67.2)	13 (68.4)	28 (66.7)	0.892
Diabetes	9 (14.8)	1 (5.3)	8 (19.0)	0.128
Coronary heart disease	18 (29.5)	5 (26.3)	13 (31.0)	0.771
Atrial fibrillation	18 (29.5)	6 (31.6)	12 (28.6)	0.811
NYHA class III/IV	50 (82.0)	15 (78.9)	35 (83.3)	0.683
**Echocardiographic assessment**				
LVEF, %	54.3 ± 12.4	53.4 ± 12.6	54.7 ± 12.4	0.707
LVEDD, mm	58.0 ± 6.3	59.0 ± 4.6	57.6 ± 6.9	0.416
Mean aortic valve gradient	9.1 ± 4.8	9.5 ± 5.6	9.0 ± 4.5	0.732
Transaortic peak velocity	195.5 ± 50.6	198.4 ± 55.5	194.3 ± 49.2	0.795
≥Moderate mitral regurgitation	22 (36.1)	8 (42.1)	14 (33.3)	0.509
Cause of regurgitation				0.424
Leaflet degeneration	54 (88.5)	16 (84.2)	38 (90.5)	
Leaflet prolapse	6 (9.8)	2 (10.5)	4 (9.5)	
Leaflet injury	1 (1.6)	1 (5.3)	0	

Data are presented as mean ± standard deviation or n (%).

STS, Society of Thoracic Surgeons; BMI, body mass index; NYHA, New York Heart Association; LVEDD, left ventricular end-diastolic diameter; LVEF, left ventricular ejection fraction.

**TABLE 2:** Anatomic characteristics of the patients.

	Total population (*n* = 61)	Severe malposition (*n* = 19)	Non/mild malposition (*n* = 42)	*P*-value
Types of aortic valve				0.588
Type 0 bicuspid	2 (3.3)	0	2 (4.8)	
Tricuspid	56 (91.8)	19 (100)	37 (88.1)	
Quadricuspid	3 (4.9)	0	3 (7.1)	
Prosthesis size				0.092
L26	13 (21.3)	2 (10.5)	11 (26.2)	
L29	30 (49.2)	8 (42.1)	22 (52.4)	
L32	18 (29.5)	9 (47.4)	9 (21.4)	
**Annulus**				
Maximum diameter, mm	27.5 ± 2.8	27.8 ± 2.0	27.3 ± 3.1	0.577
Minimum diameter, mm	21.7 ± 2.0	22.2 ± 2.0	21.4 ± 1.9	0.188
Mean diameter, mm	24.6 ± 2.2	25.0 ± 1.7	24.4 ± 2.4	0.356
Perimeter, mm	78.5 ± 7.1	79.8 ± 4.9	77.9 ± 7.8	0.316
Area, mm^2^	474.1 ± 85.5	488.6 ± 61.9	467.6 ± 94.3	0.379
**LVOT**				
Mean diameter, mm	25.5 ± 3.2	25.6 ± 2.7	25.4 ± 3.5	0.774
**STJ**				
Mean diameter, mm	33.1 ± 3.6	34.8 ± 3.4	32.3 ± 3.4	**0.009**
Height, mm	22.8 ± 4.0	24.9 ± 5.3	21.9 ± 2.9	**0.030**
AA diameter, mm	37.4 ± 3.5	38.3 ± 3.1	37.1 ± 3.7	0.228
Calcification volume, mm^3^	0 (0, 5.65)	0 (0, 21.5)	0 (0, 4.6)	0.563
Aortic root angulation, degree	55.9 ± 10.3	56.4 ± 9.7	55.7 ± 10.7	0.814
**Ratio within aortic root**				
LVOT perimeter/Annulus perimeter	1.04 ± 0.07	1.03 ± 0.07	1.04 ± 0.07	0.691
STJ diameter/Annulus diameter	1.32 ± 0.14	1.36 ± 0.15	1.30 ± 0.13	0.118
AA diameter/STJ diameter	1.14 ± 0.09	1.10 ± 0.10	1.15 ± 0.08	0.075
AA diameter/Annulus diameter	1.52 ± 0.18	1.54 ± 0.17	1.52 ± 0.18	0.744
**Ratio between aortic root and prosthesis**				
Valve oversize ratio, %	17.4 ± 6.7	18.5 ± 5.7	16.9 ± 7.2	0.385
STJ cover index, %	80.3 ± 85.8	84.0 ± 8.5	78.7 ± 8.2	**0.027**
Prosthesis crown diameter/AA diameter	1.11 ± 0.11	1.09 ± 0.08	1.12 ± 0.11	0.373

Data are presented as mean ± standard deviation, median (interquartile range), or n (%).

LVOT, left ventricular outflow tract; STJ, sinotubular junction; AA, ascending aorta.

Bold values indicates *p* < 0.05.

**TABLE 3:** Procedural characteristics and clinical outcomes.

	Total population (*n* = 61)	Severe malposition (*n* = 19)	Non/mild malposition (*n* = 42)	*P*-value
**Procedural characteristics**				
Device generation				0.785
Non-resheathable Venus-A	33 (54.1)	11 (57.9)	22 (52.4)	
Resheathable Venus-A Plus	28 (45.9)	8 (42.1)	20 (47.6)	
Reposition time(s)				0.813
Without reposition	20 (71.4)	7 (87.5)	13 (65.0)	
Once	6 (21.4)	1 (12.5)	5 (25.0)	
Twice	2 (7.1)	0	2 (10.0)	
Transfemoral approach	60 (98.4)	19 (100)	41 (97.6)	1.000
General anesthesia	60 (98.4)	18 (94.7)	42 (100)	0.311
Rapid pacing	61 (100)	19 (100)	42 (100)	NA
Post dilation	1 (1.6)	1 (5.9)	0	0.311
Implantation depth, mm	11.4 ± 7.3	19.0 ± 3.2	7.7 ± 5.7	**<0.001**
Valve-in-valve implantation	13 (21.3)	12 (63.2)	1 (2.4)	**<0.001**
Convert to open surgery	1 (1.6)	1 (5.3)	0	0.311
**Device success (at 30 days)**	45 (73.8)	4 (21.1)	41 (97.6)	**<0.001**
Technical success	48 (78.7)	7 (36.8)	41 (97.6)	**<0.001**
Mortality	2 (3.3)	1 (5.3)	1 (2.4)	0.530
Re-intervention related to device	14 (23.0)	13 (68.4)	1 (2.4)	**<0.001**
Intended valve performance	45 (73.8)	5 (26.3)	40 (95.2)	**<0.001**
MG < 20 mm Hg and PV < 3 m/s	60 (98.4)	18 (94.7)	42 (100)	0.311
No moderate or severe AR	45 (73.8)	5 (26.3)	40 (95.2)	**<0.001**
**Early safety (at 30 days)**	31 (50.8)	4 (21.1)	27 (64.3)	**0.002**
All-cause mortality	2 (3.3)	1 (5.3)	1 (2.4)	0.530
Stroke	2 (3.3)	0	2 (4.8)	1.000
Major bleeding	6 (9.8)	3 (15.8)	3 (7.1)	0.364
Access or cardiac complication	2 (3.3)	1 (5.3)	1 (2.4)	0.530
Acute kidney injury	0	0	0	NA
Moderate or severe AR	16 (26.2)	14 (73.7)	2 (4.8)	**<0.001**
New PPM	12 (19.7)	3 (15.8)	9 (21.4)	0.602
Re-intervention related to device	14 (23.0)	13 (68.4)	1 (2.4)	**<0.001**
**Other 30-day clinical outcomes**				
MG, mmHg, at 30-day	8.0 ± 4.0	7.8 ± 3.7	8.1 ± 4.1	0.807
PV, cm/s, at 30-day	192.8 ± 41.0	199.9 ± 44.0	189.7 ± 39.7	0.404
≥mild perivalvular leakage	29 (47.5)	19 (100)	10 (23.8)	**<0.001**
Impaired AML movement				**<0.001**
Significant impaired	14 (23.0)	10 (52.6)	4 (9.5)	
Not impaired	25 (41.0)	1 (5.3)	24 (57.1)	
Uncertain/Unkown	22 (36.1)	8 (42.1)	14 (33.3)	
NYHA class III/IV at 30 days	8 (13.1)	6 (31.6)	2 (4.8)	**0.006**
Re-hospitalization due to HF	4 (6.6)	4 (21.1)	0	**0.002**
All cause re-hospitalization	5 (8.2)	4 (21.1)	1 (2.4)	**0.018**

Data are presented as mean ± standard deviation or n (%).

NA, not applicable; MG, mean gradient; PV, peak velocity; PPM, permanent pacemaker implantation; HF, heart failure; AML, anterior mitral leaflet; NYHA, New York Heart Association.

Bold values indicates *p* < 0.05.

## Results

Among 61 patients, 33 underwent TAVR with Venus-A and 28 with Venus-A Plus prosthesis valve. Sixty approaches were transfemoral and 1 was performed using the transcarotid approach. The non-/mid malposition group included 20 optimal position cases and 22 mild malposition cases. Among the 19 severe malposition cases, obvious valve migration toward the left ventricle causing residual stenosis or more than moderate paravalvular regurgitation was identified in 17 patients; in 15 patients (88.2%), it occurred during the TAVR procedure, and in 2 patients (11.8%), it occurred later. Both patients with delayed migration received successful single-valve implantation with acceptable hemodynamics immediately after the TAVR procedure. One complained of dyspnea 3 days after the TAVR procedure, and valve migration toward the ventricle was confirmed by transthoracic echocardiography, accompanied by new-onset severe perivalvular leakage. Subsequently, the patient received valve-in-valve TAVR. The other patient was asymptomatic at his 30-day follow-up, while transthoracic echocardiography revealed prosthesis much deeper than before discharge (implantation depth changed from 15.8 to 21.5 mm), followed by perivalvular leakage, which progressed from mild to severe. This patient refused invasive treatment and insisted on medication. As shown in Table 1, the baseline characteristics such as age, body mass index, and echocardiographic assessment parameters were comparable between the two groups (all *p* > 0.05).

The anatomic characteristics are shown in Table 2. According to the CT parameters, the study population was without calcification in aortic cusps. The diameter and height of STJ of the severe malposition group were both significantly greater compared with the non-/mild malposition group (both *p* < 0.05). Furthermore, the severe malposition group was associated with a greater STJCI (*p* = 0.027). Procedural characteristics and 30-day clinical outcomes are listed in Table 3. The proportion of resheathable valve application showed no significant difference between the two groups; however, the implantation depth was significantly deeper in patients with severe prosthesis malposition (19.0 ± 3.2 vs. 7.7 ± 5.7 mm, *p* < 0.001). A total of 63.2% (12/19) cases received additional valve implantation in the severe malposition group and one case (2.4%) in the non-/mild malposition group. All the valve-in-valve procedures were implanted with the same size Venus-A prosthesis as the first valve. One patient received open surgery 2 days after the TAVR procedure due to severe residual AR and new-onset moderate stenosis of the mitral valve after very deep implantation. One patient received post-dilation because of the high residual transvalvular gradient after severe ventricular migration of the first valve.

Regarding 30-day clinical outcomes, there were no significant differences in mortality, permanent pacemaker implantation, major vascular complication, and major bleeding between the two groups, while the device success rate (21.1 vs. 97.6%, *p* < 0.001) and early safety rate (21.1 vs. 64.3%, *p* < 0.001) were significantly lower in the severe malposition group. These differences were mainly driven by residual moderate or more AR and reintervention (valve in valve TAVR). Furthermore, the incidence of impaired AML movement was higher in the severe malposition group (52.6 vs. 9.5%, *p* < 0.001). At 30-day follow up, the proportion of New York Heart Association (NYHA) class III/IV (*p* = 0.006) and incidence of rehospitalization due to heart failure (*p* = 0.002) or all-cause rehospitalization (*p* = 0.018) were higher in the severe malposition group. One patient was rehospitalized in the non-/mild malposition group due to major gastrointestinal bleeding.

Table 4 shows the results of the univariate analyses of predictors of severe prosthesis malposition. The diameter (OR = 1.23, *p* = 0.003) and height (OR = 1.24, *p* = 0.017) of STJ were positively correlated with severe prosthesis malposition, as well as STJCI (OR = 1.08, *p* = 0.032). Large prosthesis (L32 size) implantation has the tendency of severe malposition regarding the marginal statistical significance (OR = 5.50, *p* = 0.059). As shown in Figure 2B, the AUC was 0.72 (95% CI 0.58–0.85, *p* = 0.008) for STJ diameter, 0.70 (95% CI 0.55–0.86, *p* = 0.012) for STJ height, and 0.69 (95% CI 0.55–0.83, *p* = 0.017) for STJCI, respectively. The cutoff value was 33.2 mm for STJ diameter (sensitivity was 84.2% and specificity was 65.8%), 24.1 mm (sensitivity was 57.9% and specificity was 87.8%) for STJ height, and 81.0% (sensitivity was 68.4% and specificity was 68.3%) for STJCI, respectively. The three factors correlated with each other significantly (correlation coefficient 0.42–0.88, all *p* < 0.05, Supplementary Table 3).

**TABLE 4:** Logistic regression analysis of predictors of severe valve malposition.

	Univariate logistic regression analysis		
Parameter	OR	95% CI	*P*-value
Male gender (1 = yes, 0 = no)	3.098	0.879–10.913	0.078
Annulus perimeter, mm	1.040	0.963–1.123	0.318
Valve oversize ratio, %	1.040	0.953–1.135	0.380
Valve oversize ratio > 20% or <10% (1 = yes, 0 = no)	1.200	0.404–3.563	0.743
LVOT mean diameter, mm	1.025	0.868–1.210	0.769
LVOT perimeter/Annulus perimeter	0.198	0–495.052	0.685
STJ mean diameter, mm	1.234	1.039–1.467	**0.017**
STJ height, mm	1.237	1.039–1.473	**0.017**
STJCI, %	1.080	1.007–1.159	**0.032**
Ascending aorta diameter, mm	1.103	0.941–1.292	0.226
Ascending aorta diameter/STJ*100	0.942	0.880–1.007	0.081
**Implanted prosthesis size**			
**L26 (Reference)**			
L29	2.000	0.362–11.060	0.427
L32	5.500	0.939–32.205	0.059
Venus A plus Implication (1 = yes, 0 = no)	0.800	0.268–2.388	0.689

LVOT, left ventricular outflow tract; STJCI, sinotubular junction.

Bold values indicate *p* < 0.05.

## Discussion

In this study, we investigated how the anatomic factors affected TAVR procedure performance among patients with NAVR from four large volume centers in China. Our results showed that the incidence of severe prosthesis malposition/embolization was 32.3% (20/62) following TAVR with self-expandable Venus-A Valve among patients with NAVR, most (19/20) of whom were with downward migration in the ventricular direction. Furthermore, the STJ diameter > 33.2 mm, height > 24.1 mm, and STJCI > 81.0% could predict severe prosthesis malposition. Finally, severe prosthesis malposition was associated with worse early safety, as well as impaired AML movement, heart failure, and rehospitalization at a 30-day follow-up.

To the best of our knowledge, this is the first report to explore anatomic risk factors of prosthesis malposition among patients with NAVR who received self-expandable TAVR. Previous studies reported a low incidence (∼3%) of prosthesis malposition among the aortic valve stenosis population (12–14), while it reached up to approximately 20% in patients with NAVR who underwent TAVR with self-expandable devices (2, 15). This proportion is close to our data, partially because these studies used morphologically similar CoreValve prostheses (Medtronic, Minneapolis, USA) (2, 15). Valve migration or embolization may occur upward in the aortic direction and downward in the ventricular direction (13, 15). De Backer et al. (2) found that among patients with NAVR who underwent TAVR with CoreValve and Evolut R prosthesis, 13 out of 40 sizing error cases occurred in the ventricular direction valve migration and 6 were up toward the aortic direction. In the current study, 95.0% (19/20) of severe prosthesis malpositions were toward the ventricular direction, and there was only one case of valve embolization toward the ascending aortic aorta. Consistent with our findings, Yin et al. (6) reported that the malposition rate of the CoreValve device in patients with pure NAVR was 62%, all of which were caused by too-low implantation. In another study involving Chinese patients with aortic stenosis who underwent TAVR with Venus-A Valve, all valve malpositions were toward the ventricular direction (9), which may be partly explained by greater radial force at the bottom section of the Venus A-valve that could enhance the downward pushing force during delivering the prosthesis (16). Another possible reason is that the operator deliberately selected a somewhat deeper position to avoid prosthesis embolization to the aorta (6). Nevertheless, in a patient with the upward valve migration, the AA and STJ were quite slender, and the strong interaction between the prosthesis crown and STJ/AA was deemed to generate the upward force, which ultimately led to upward skipping of the prosthesis valve. Given the contrasting anatomic features of aortic root among patients with upward and downward migration, there was only one case in the aortic direction group; therefore, only 19 patients with downward migration were included in the final analysis.

As the absence of calcification hinders prosthesis anchoring and increases the risk of valve migration, careful evaluation of the aortic root anatomy for selecting suitable patients is of great importance. It is generally believed that suitable candidates should not have too large an annulus. Also, at least a 15–20% device oversize ratio is recommended (6, 11, 17). In the present study, the mean diameter of the annulus was 24.6 mm and the oversize ratio was 17.4%, which certainly represented a “selected” patient population. In fact, more patients with severe NAVR were considered for TAVR but were turned down because of anatomy or other reasons (15). Yet, even in this selected population, the rate of severe prosthesis malposition remained at 32.3%. In particular, among those who received 32-mm valve implantation, the rate was as high as 50.0% (9/18). Undeniably, a larger annulus could hardly provide enough supporting force to prevent the downward migration of the prosthesis. Based on our data, large prosthesis (32 mm) implantation has the tendency of severe malposition according to the marginal statistical significance in logistic regression (OR = 5.50, *p* = 0.059). Large cohort research could be performed to explore a threshold of annulus size for predicting severe malposition in the future. Moreover, the interaction of STJ and prosthesis crown could also provide force against downward valve migration, thus explaining why smaller STJCI and STJ diameter were associated with greater force to prevent ventricular migration, while too small STJCI might lead to upward valve migration in the context of the slender AA. To the best of our knowledge, this study first reported the ratio of STJ size and prosthesis crown (STJCI) related to prosthesis malposition. In view of the intrinsic correlation of STJ diameter, STJ height, and STJCI, we prefer to choose an index that combines valve size and STJ morphology, thus we further identified the best threshold value of STJCI > 81.0% as a predictor of severe prosthesis malposition. Because only the Venus-A Valve was used in this study, the predictive performance of these indicators should be further evaluated in different prosthesis heart valves. It is worth mentioning that in Li’s study (9), a “conical LVOT” was associated with deep implantation, while we did not detect the difference in LVOT perimeter/annulus perimeter between the malposition and non-malposition group. It is possible that in the absence of an adequate anchor the prosthesis valve has a tendency to move down during releasing, and the upward force of STJ against valve migration was significantly stronger than that of LVOT in patients with pure NAVR; hence, the effect of LVOT was covered, especially in this small sample.

Surprisingly, we noticed that the new-generation Venus-A Plus application did not reduce the incidence of severe malposition (42.1 vs. 57.9%, *p* = 0.785), which is inconsistent with previous studies (2, 4, 6, 18). The reason remains speculative, while a possible explanation may be that the stronger radial force of Venus-A Valve enhanced the downward migration tendency, even after repositioning with a resheathable delivery system. Also, the self-expanding valve was “self-adaptive” to match the best position within the native aortic root that could provide the most appropriate force against ventricular direction migration. In a sense, the uselessness of resheathable Venus-A Plus to minimize malposition introduced more strict requirements for patient selection before making final treatment strategy decisions.

As for the clinical impact of prosthesis malposition, we found no significant difference in mortality, need for permanent pacemaker implantation, or other VARC-3 defined endpoint events between the two groups at 30-day follow-up, while the device success and early safety were significantly lower in the severe malposition group. Moreover, the rates of heart failure and rehospitalization (mainly driven by heart failure) were higher in the severe malposition group. Residual AR may be responsible for the worse heart function. Besides, too-deep prosthesis implantation could also impair adequate AML movement (52.6 vs. 9.5%, *p* < 0.001), thus resulting in worse hemodynamics and cardiac function (19, 20).

### Study limitations

There are several limitations in the present study. First, given the relatively small sample and retrospective observational design, we must be cautious when drawing firm conclusions due to unmeasured confounders. Second, we only discussed anatomic risk factors when implanting the Venus-A Valve in this study, while various other reasons may be responsible for prosthesis malposition, such as improper post-dilation, sizing errors, and fast-rate pacing failures. Other limitations are patient selection bias, short follow-up duration, and no independent core laboratory or adjudication of clinical events. Consequently, further studies aiming to explore more predictors on a larger scale using different types of prosthesis valves are needed to verify reported results.

## Conclusion

Our data suggest that larger and higher STJ and greater STJ to valve crown diameter ratio (STJCI > 81.0%) are potential predictors of severe prosthesis malposition in patients with NAVR who underwent TAVR with Venus-A prosthesis valve.

## Data availability statement

The raw data supporting the conclusions of this article will be made available by the authors, without undue reservation.

## Ethics statement

The studies involving human participants were reviewed and approved by Research Ethics Committee of The Second Affiliated Hospital of Army Military Medical University. Written informed consent for participation was not required for this study in accordance with the national legislation and the institutional requirements.

## Author contributions

YW and SY contributed equally to study design, data acquisition, statistical analysis, and drafted the manuscript. JJ approved the submission of the final version. DQ, JL, ZF, WC, XL, TL, YZ, and HX contributed greatly to data collection and the revision of the manuscript. All authors contributed to the article and approved the submitted version.
