# Supplementary material for: Anatomic predictor of severe prosthesis malposition following transcatheter aortic valve replacement with self- expandable Venus-A Valve among pure aortic regurgitation: A multicenter retrospective study
Source: Front Cardiovasc Med. 2022 Dec 8;9:1002071. doi: 10.3389/fcvm.2022.1002071 (PMC9775278; doi:10.3389/fcvm.2022.1002071)
Supplement: Supplementary file 1 [file Data_Sheet_1.docx]

**Supplementary Table 1 Anatomic characteristics across the groups**

|  | **Severe Malposition**  **(n=19)** | **Mild Malposition**  **(n=22)** | **Optimal implantation**  **(n=20)** | ***P* Value** |
| --- | --- | --- | --- | --- |
| ***Types of aortic valve*** |  |  |  | 0.843 |
| Type 0 Bicuspid | 0 | 1 (4.5) | 1 (5.0) |  |
| Tricuspid | 19 (100) | 19 (86.4) | 18 (90.0) |  |
| Quadricuspid | 0 | 2 (9.1) | 1 (5.0) |  |
| ***Prosthesis size*** |  |  |  | 0.153 |
| L26 | 2 (10.5) | 4 (18.2) | 7 (35.0) |  |
| L29 | 8 (42.1) | 12 (54.5) | 10 (50.0) |  |
| L32 | 9 (47.4) | 6 (27.3) | 3 (15.0) |  |
| ***Annulus*** |  |  |  |  |
| Maximum Diameter, mm | 27.8±2.0 | 27.7±3.7 | 27.0±2.3 | 0.658 |
| Minimum Diameter, mm | 22.2±2.0 | 21.8±2.0 | 21.0±1.8 | 0.187 |
| Mean Diameter, mm | 25.0±1.7 | 24.8±2.8 | 24.0±1.9 | 0.376 |
| Perimeter, mm | 79.8±4.9 | 79.0±8.7 | 76.6±6.7 | 0.334 |
| Area, mm^2^ | 488.6±61.9 | 482.0±108.4 | 451.7±75.2 | 0.354 |
| ***LVOT*** Mean Diameter, mm | 25.6±2.7 | 26.1±3.2 | 24.6±3.6 | 0.291 |
| ***STJ*** |  |  |  |  |
| Mean Diameter, mm | 34.8±3.4 | 32.0±3.2 | 32.6±3.6 | **0.031** |
| Height, mm | 24.9±5.3 | 22.1±3.2 | 21.6±2.5 | **0.023** |
| ***AA*** diameter, mm | 38.3±3.1 | 36.7±3.9 | 37.5±3.6 | 0.381 |
| ***Calcification volume***, mm^3^ | 0 (0, 21.5) | 0 (0, 14.0) | 0 (0, 0.15) | 0.563 |
| ***Aortic root angulation***, degree | 56.4±9.7 | 57.6±8.1 | 53.6±12.8 | 0.443 |
| ***Ratio within aortic root*** |  |  |  |  |
| LVOT perimeter/ Annulus perimeter | 1.03±0.07 | 1.05±0.06 | 1.02±0.08 | 0.264 |
| STJ diameter/ Annulus diameter | 1.36±0.15 | 1.27±0.14 | 1.34±0.11 | 0.095 |
| AA diameter/STJ diameter | 1.10±0.10 | 1.15±0.08 | 1.15±0.09 | 0.205 |
| AA diameter/Annulus diameter | 1.54±0.17 | 1.49±0.19 | 1.56±0.17 | 0.428 |
| ***Ratio between aortic root and prosthesis*** |  |  |  |  |
| Valve oversize ratio,% | 18.5±5.7 | 17.0±7.6 | 16.8±7.0 | 0.684 |
| STJ cover index, % | 84.0±8.5 | 80.0±7.6 | 77.5±8.7 | **0.047** |
| Prosthesis crown diameter/AA diameter | 1.09±0.08 | 1.13±0.11 | 1.10±0.11 | 0.443 |

Data are presented as mean ± standard deviation, median (interquartile range), or n (%).

LVOT, Left Ventricular Outflow Tract; STJ, SinoTubular Junction. AA, Ascending aorta.

Bold values indicates *p*<0.05.

**Supplementary Table 2 Procedural data and clinical outcomes across the groups**

|  | **Severe Malposition**  **(n=19)** | **Mild Malposition**  **(n=22)** | **Optimal implantation**  **(n=20)** | ***P* Value** |
| --- | --- | --- | --- | --- |
| ***Procedural characteristics*** |  |  |  | 0.876 |
| Device generation |  |  |  |  |
| Non-resheathable Venus-A | 11 (57.9) | 11 (50.0) | 11 (55.0) |  |
| Resheathable Venus-A Plus | 8 (42.1) | 11 (50.0) | 9 (45.0) |  |
| Transfemoral Approach | 19 (100) | 21 (95.5) | 20 (100) | 1.000 |
| General anesthesia | 18 (94.7) | 22 (100) | 20 (100) | 0.311 |
| Rapid pacing | 19 (100) | 22 (100) | 20 (100) | NA |
| Post dilation | 1 (5.9) | 0 | 0 | 0.311 |
| Implantation depth, mm | 19.0±3.2 | 12.1±1.6 | 3.7±2.8 | **<0.001** |
| Valve-in-valve implantation | 12 (63.2) | 1 (4.5) | 0 | **<0.001** |
| Convert to open surgery | 1 (5.3) | 0 | 0 | 0.311 |
| ***Device success (at 30 days)*** | 4 (21.1) | 21 (95.5) | 20 (100) | **<0.001** |
| Technical success | 7 (36.8) | 21 (95.5) | 20 (100) | **<0.001** |
| Mortality | 1 (5.3) | 0 | 1 (5.0) | 0.531 |
| Re-intervention related to device | 13 (68.4) | 1 (4.5) | 0 | **<0.001** |
| Intended valve performance | 5 (26.3) | 20 (90.9) | 20 (100) | **<0.001** |
| MG<20 mm Hg and PV<3 m/s | 18 (94.7) | 22 (100) | 20 (100) | 0.311 |
| No moderate or severe AR | 5 (26.3) | 20 (90.9) | 20 (100) | **<0.001** |
| ***Early safety (at 30 days)*** | 4 (21.1) | 12 (54.5) | 15 (75.0) | **0.003** |
| All-cause mortality | 1 (5.3) | 0 | 1 (5.0) | 0.531 |
| Stroke | 0 | 2 (9.1) | 0 | 0.323 |
| Major Bleeding | 3 (15.8) | 3 (13.6) | 0 | 0.077 |
| Access or cardiac complication | 1 (5.3) | 0 | 1 (5.0) | 0.531 |
| Acute kidney injury | 0 | 0 | 0 | NA |
| Moderate or severe AR | 14 (73.7) | 2 (9.1) | 0 | **<0.001** |
| New PPM | 3 (15.8) | 5 (22.7) | 4 (20.0) | 0.853 |
| Re-intervention related to device | 13 (68.4) | 1 (4.5) | 0 | **<0.001** |
| ***Other 30-day clinical Outcomes*** |  |  |  |  |
| MG, mmHg, at 30-day | 7.8±3.7 | 7.8±3.4 | 7.9±4.7 | 0.998 |
| PV, cm/s, at 30-day | 199.9±44.0 | 189.6±31.2 | 185.8±50.1 | 0.586 |
| ≥ mild perivalvular leakage | 19 (100) | 7 (31.8) | 3 (15.0) | **<0.001** |
| Impaired AML movement |  |  |  | **<0.001** |
| Significant impaired | 10 (52.6) | 4 (18.2) | 0 |  |
| Not impaired | 1 (5.3) | 7 (31.8) | 17 (85.0) |  |
| Uncertain/Unkown | 8 (42.1) | 11 (50.0) | 3 (15.0) |  |
| NYHA class III/IV at 30d | 6 (31.6) | 2 (9.1) | 0 | **0.006** |
| Re-hospitalization due to HF | 4 (21.1) | 0 | 0 | **0.007** |
| All cause re-hospitalization | 4 (21.1) | 1 (4.5) | 0 | **0.032** |

Data are presented as mean ± standard deviation or n (%). NA, Not Applicable.

MG, Mean Gradient. PV, Peak Velocity.

PPM, Permanent Pacemaker Implantation; HF, Heart Failure; AML, anterior mitral leaflet.

NYHA, New York Heart Association.

Bold values indicates *p*<0.05.

**Supplementary table 3 Pearson correlation coefficients of the 3 factors**

|  |  | **STJCI** | **STJ diameter** | **STJ height** |
| --- | --- | --- | --- | --- |
| **STJCI** | Correlation coefficient | 1 | 0.881 | 0.422 |
|  | *P* value |  | <0.001 | 0.001 |
| **STJ diameter** | Correlation coefficient |  |  | 0.430 |
|  | *P* value |  |  | 0.001 |

STJCI, sinotubular junction cover index
