# Supplementary figures and images for: Anatomic predictor of severe prosthesis malposition following transcatheter aortic valve replacement with self- expandable Venus-A Valve among pure aortic regurgitation: A multicenter retrospective study
Source: Front Cardiovasc Med. 2022 Dec 8;9:1002071. doi: 10.3389/fcvm.2022.1002071 (PMC9775278; doi:10.3389/fcvm.2022.1002071)

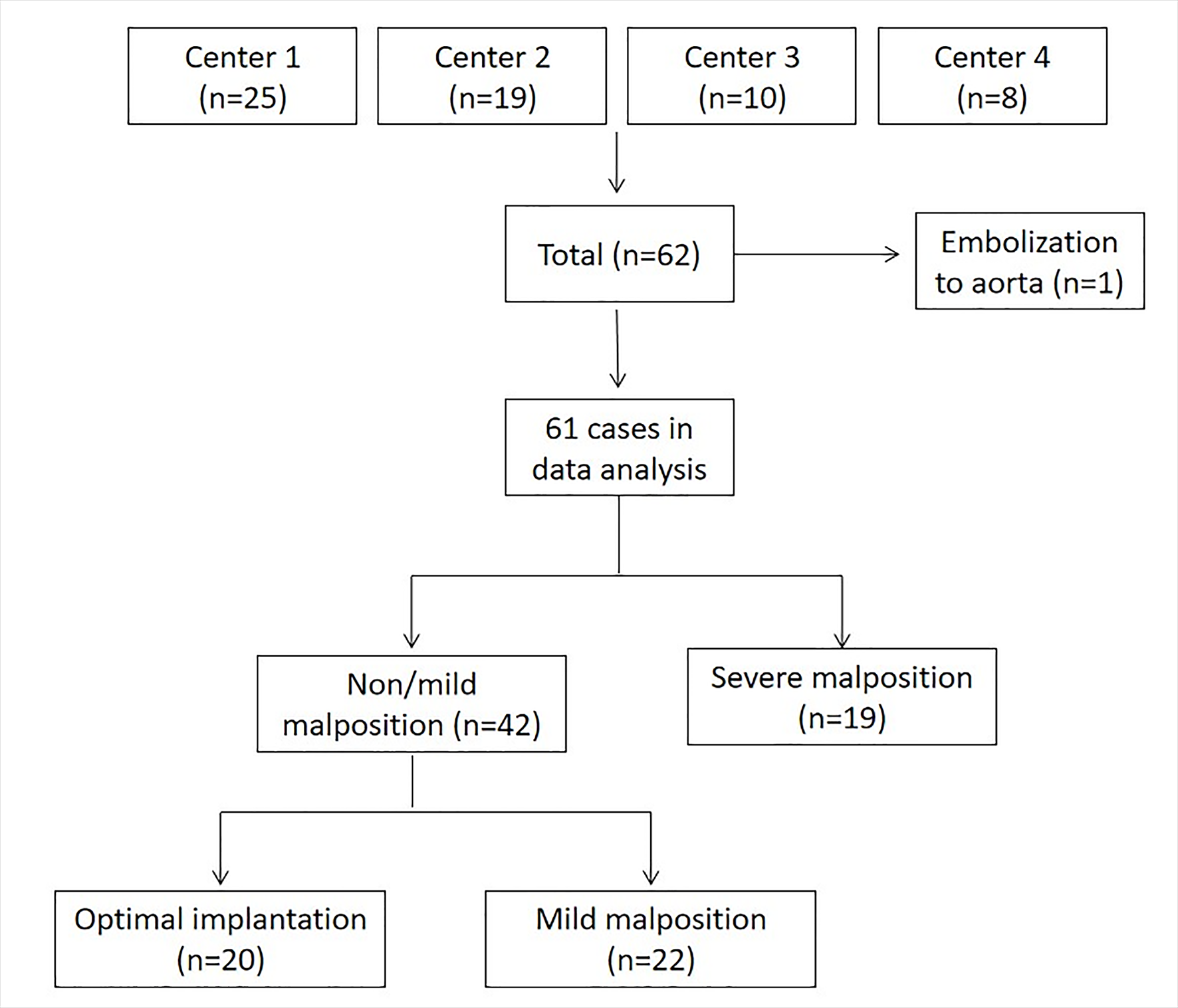

Supplement: Supplementary file 2 [file Image_1.TIF]

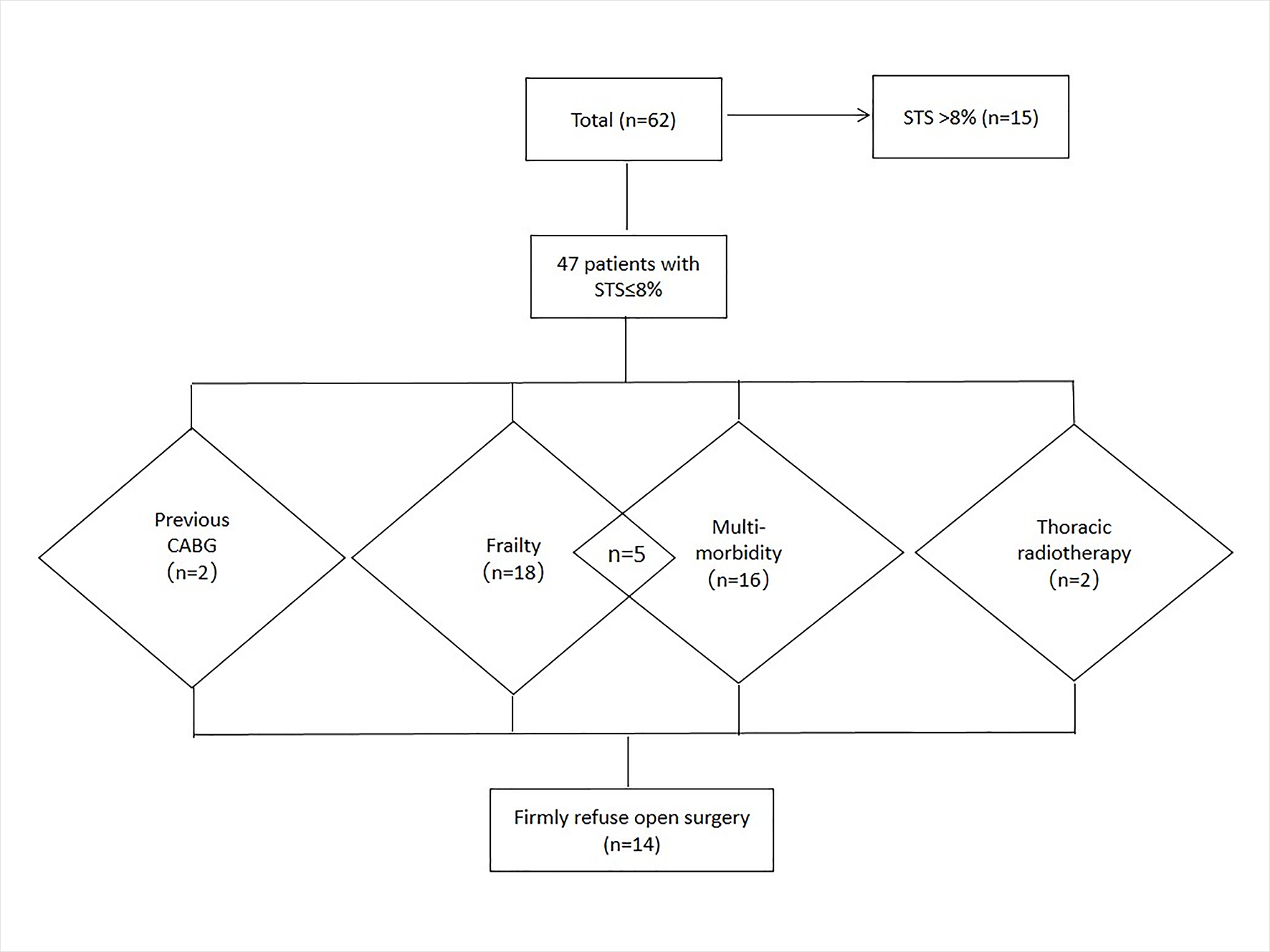

Supplement: Supplementary file 3 [file Image_2.TIF]
